# Vaidya geometries and scalar fields with null gradients

**DOI:** 10.1140/epjc/s10052-021-09040-9

**Published:** 2021-03-15

**Authors:** Valerio Faraoni, Andrea Giusti, Bardia H. Fahim

**Affiliations:** Department of Physics and Astronomy, Bishop’s University, 2600 College Street, Sherbrooke, Québec J1M 1Z7 Canada

## Abstract

Since, in Einstein gravity, a massless scalar field with lightlike gradient behaves as a null dust, one could expect that it can act as the matter source of Vaidya geometries. We show that this is impossible because the Klein–Gordon equation forces the null geodesic congruence tangent to the scalar field gradient to have zero expansion, contradicting a basic property of Vaidya solutions. By contrast, exact plane waves travelling at light speed and sourced by a scalar field acting as a null dust are possible.

## Null dust and Vaidya’s spacetime

The Vaidya solutions of the Einstein equations [1, 2]

1 $$\begin{aligned} R_{ab}-\frac{1}{2} \, g_{ab}R =8\pi T_{ab} \end{aligned}$$

(where $R_{ab}$ is the Ricci tensor of the metric $g_{ab}$, *R* is its trace, and $T_{ab}$ is the matter energy-momentum tensor)^1^ are used to study gravitational collapse, the formation and evolution of event and apparent horizons, and as toy models of evaporating black holes. Vaidya geometries are also useful in string theory and holography (*e.g.*, [4, 5]). The source of a Vaidya geometry is a null dust, described by the stress-energy tensor

2 $$\begin{aligned} T_{ab}= \rho \, \ell _a \ell _b \,, \end{aligned}$$

where $\rho$ is the dust density and the four-vector $\ell ^a$ is null and geodesic,

3 $$\begin{aligned} \ell ^c \ell _c = 0 \,, \;\;\;\;\;\;\;\;\;\; \ell ^c \nabla _c \ell ^a= - \left( \ell ^b \nabla _b \ln \rho + \nabla ^b \ell _b \right) \ell _a \,, \end{aligned}$$

as follows from the conservation equation $\nabla ^b T_{ab}=0$ (see Ref. [6] for a review on the null dust). Contrary to timelike geodesics, for which the normalization selects the proper time as the parameter along the curve, for null geodesics the null normalization of the four-tangent does not impose affine parametrization. As a consequence, in general, the covariant conservation equation $\nabla ^b T_{ab} =0$ for the stress-energy tensor (2) gives non-affinely parametrized geodesics. Since the components $\ell ^{\mu }$ of a null vector can be reparametrized as $\ell ^{\mu }\rightarrow {\bar{\ell }}^{\mu } = f \ell ^{\mu }$ (where $f>0$ is a function of the coordinates) without changing its normalization, a representation can be chosen in which $\rho$ is unity by choosing $f=\sqrt{\rho }$, but the corresponding null geodesics followed by the null dust are not affinely parametrized [6].

A null dust appears frequently in classical and quantum gravity, in Vaidya spacetimes [1, 2], *pp*-waves [6–8], Robinson-Trautman geometries [9], twisting solutions of the Einstein–Maxwell equations [9–11], in studies of classical and quantum gravitational collapse, horizon formation, mass inflation [12–21], black hole evaporation [22–24], and canonical Hamiltonians [4, 6, 25]. Colliding scalar field-null dust solutions were studied in [26, 27]. A null dust is interpreted as a coherent zero rest mass field that propagates at light speed in the null direction $\ell ^a$, in the geometric optics limit. In this sense, null dust is more closely related to fundamental fields than its timelike counterpart [6], which explains its widespread use as a matter source in the investigation of fundamental questions in gravity.

An outgoing Vaidya solution [1, 2] (see also [28, 29]) is given by the line element

4 $$\begin{aligned} ds^2=-\left( 1-\frac{2m(u)}{r} \right) du^2 -2dudr +r^2 d\Omega _{(2)}^2 \,, \end{aligned}$$

where *u* is a retarded null coordinate, *m*(*u*) is a regular mass function, and $d\Omega _{(2)}^2 \equiv d\vartheta ^2 +\sin ^2 \vartheta \, d\varphi ^2$ is the line element on the unit 2-sphere. The only non-vanishing component of the Ricci tensor is

5 $$\begin{aligned} R_{uu} = -\, \frac{2m'(u)}{r^2} \,, \end{aligned}$$

where a prime denotes differentiation with respect to *u*. The stress-energy tensor of the null dust sourcing the Vaidya geometry is

6 $$\begin{aligned} T_{ab}= -\, \frac{m'(u)}{4\pi r^2} \, \ell _a \ell _b \,, \end{aligned}$$

where the vector $\ell ^a$ is null and geodesic. For the outgoing Vaidya metric, it is $\ell _a=-\partial _a u$ [28, 29].

## Scalar field as a null dust and the impossibility of scalar field-sourced Vaidya geometries

Scalar field theory is the simplest field theory and scalar fields are ubiquitous in particle physics, cosmology, scalar-tensor gravity, and in models of classical and quantum gravity in general [3, 30–37]. A null dust can be achieved by means of a scalar field $\phi$ with null gradient $\nabla ^c \phi \nabla _c \phi =0$ [6, 38]. This result follows from a specification of the dynamics. For example, a Klein-Gordon scalar field with null gradient $\nabla _c \phi$ constitutes an imperfect fluid of type II in the Hawking-Ellis classification [39]. Imposing the (weak, or strong, or dominant) energy condition, forces a scalar potential $V(\phi )$ that could potentially be present to vanish identically and this type II fluid becomes a null dust, as discussed in detail in Ref. [40].^2^ (Indeed, the condition $V=0$ is satisfied in the stress-energy tensor (8) below.)

It is well known [42–44] that a scalar field $\phi$ with timelike gradient, $\nabla ^c\phi \nabla _c \phi <0$, is equivalent to an irrotational perfect fluid with four-velocity

7 $$\begin{aligned} u_c= \frac{ \nabla _c\phi }{ \sqrt{ -\nabla ^a\phi \nabla _a\phi }} \,. \end{aligned}$$

Scalar fields with spacelike gradients are less interesting and seldom considered from this point of view [45]. However, recently Ref. [40] has proposed an interesting alternative interpretation as a superposition of a left-going (or, in spherical symmetry, incoming) and a right-going (in spherical symmetry, outgoing) null dust with an additional perfect fluid. In general, a certain geometry solving the Einstein equations can be attributed to more than one source (see [41] for a discussion in the context of scalar fields).

There remains the case $\nabla ^c\phi \nabla _c \phi =0$: the stress-energy tensor of a massless, minimally coupled, scalar field $\phi$ is simply

8 $$\begin{aligned} T_{ab}^{(\phi )} = \nabla _a \phi \nabla _b\phi -\frac{1}{2} \, g_{ab} \nabla ^c \phi \nabla _c \phi \,. \end{aligned}$$

The covariant conservation equation $\nabla ^b T_{ab}^{(\phi )} =0$ for the stress-energy tensor (8) gives the Klein-Gordon equation

9 $$\begin{aligned} g^{ab} \nabla _a\nabla _b \phi \equiv \Box \phi =0 \,. \end{aligned}$$

The structure of the scalar field stress-energy tensor (8) matches that of the $T_{ab}$ of a null dust if $\ell _a \equiv \nabla _a \phi$ is lightlike. Because $\ell ^a$ is a gradient, only an irrotational dust can be reproduced this way. Now, the divergence of $\ell ^a$ is

10 $$\begin{aligned} \nabla ^c \ell _c = \nabla ^c \nabla _c \phi \equiv \Box \phi =0 \end{aligned}$$

by virtue of the Klein–Gordon equation of motion for $\phi$. The covariant conservation equation $\nabla ^b T_{ab}=0$ satisfied by any dust, including the null dust with $\rho =1$, gives

11 $$\begin{aligned} {\bar{\ell }}^b \nabla _b {\bar{\ell }}_c = -\left( \nabla ^a {\bar{\ell }}_a \right) {\bar{\ell }}_c \end{aligned}$$

which is the non-affinely parametrized geodesic equation. However, since $\Box \phi =0$, the right hand side vanishes and ${\bar{\ell }}^a$ must be null and tangent to

affinely-parametrized null geodesics.

As noted in the previous section, since a null vector is defined up to a multiplicative (positive) factor, one could choose a different parametrization of the null dust in which its stress-energy tensor is instead written as $T_{ab}=\rho \, \ell _a \ell _b$ [6, 38]. For a general null dust, the covariant conservation equation $\nabla ^b T_{ab}=0$ gives non-affinely parametrized geodesics [6].

Now consider the congruence of null geodesics tangent to the null vector $\ell ^a$ (outgoing null geodesics for the geometry (4), or ingoing null geodesics for the geometry (15)). Let us focus on the outgoing Vaidya spacetime (4) first: by comparing the relation $\ell _a=-\nabla _a u$ valid in Vaidya’s geometry with $\ell _a=\nabla _a \phi$ specifying that the null dust is generated by a scalar field, we obtain (apart from an irrelevant additive integration constant) $\phi =-u$, so the scalar field is outgoing at the speed of light. However, this result is in contradiction with the Klein–Gordon equation $\Box \phi =0$ because^3^

12 $$\begin{aligned} \Box \phi= & {} \nabla ^c \ell _c = - \nabla ^c \nabla _c u \nonumber \\= & {} -\frac{1}{\sqrt{-g}} \, \partial _{\mu } \left( \sqrt{-g} \, g^{\mu \nu } \partial _{\nu } u \right) \nonumber \\= & {} - \frac{1}{\sqrt{-g}} \, \partial _{\mu } \left( \sqrt{-g} \, g^{\mu u} \right) \nonumber \\= & {} - \frac{1}{ r^2 \sin \vartheta } \, \partial _r \left( r^2 \sin \vartheta \, g^{ur} \right) = \frac{2}{r} \,, \end{aligned}$$

where we used the fact that

13 $$\begin{aligned} \left( g^{\mu \nu } \right) = \left( \begin{array}{cccc} 0 &{} -1 &{} 0 &{} 0 \\ -1 &{} \left( 1-\frac{2m}{r}\right) &{} 0 &{} 0 \\ 0 &{} 0 &{} \frac{1}{r^2} &{} 0 \\ 0 &{} 0 &{} 0 &{} \frac{1}{r^2 \sin \vartheta } \\ \end{array} \right) \end{aligned}$$

and $\sqrt{-g}=r^2 \sin \vartheta$. Clearly, $\Box \phi = 2/r \ne 0$ and the Klein–Gordon equation cannot be satisfied. Although a scalar field with lightlike gradient is a null dust realization, a scalar field-sourced outgoing Vaidya geometry is impossible. More generally, if one considers radial outgoing and ingoing null geodesics with tangents $\ell ^a$ and $n^a$ in the outgoing Vaidya geometry (4), their expansions are easily found to be

14 $$\begin{aligned} \theta _{(\ell )}= \nabla _c \ell ^c = \frac{2}{r} \,, \;\;\;\;\;\;\;\;\;\;\; \theta _{(n)}= \frac{2m(u)-r}{r^2} \,. \end{aligned}$$

Clearly, both expansions are non-vanishing for $r>2m$. Put in other words, the congruence of outgoing radial null geodesics that is deemed to have $\ell ^c=\nabla ^c \phi$ as its four-tangent field is twist-free (because $\ell ^c$ is a gradient), shear-free (because of spherical symmetry), but cannot be also expansion-free in this geometry.

The same argument, adapted to the replacement $u \rightarrow v$, demonstrates the impossibility of ingoing Vaidya solutions of the form

15 $$\begin{aligned} ds^2=-\left( 1-\frac{2m(v)}{r} \right) dv^2 +2dvdr +r^2 d\Omega _{(2)}^2 \end{aligned}$$

(where *v* is the advanced null coordinate) sourced by a scalar field, that would have to be $\phi =-v$.

It is possible to obtain analytic solutions of the Einstein equations (1) travelling at the speed of light and sourced by scalar fields acting as a null dust, provided that the expansions of the associated null rays are allowed to vanish. As an example, consider an exact plane wave sourced by a scalar field with null four-gradient. This plane wave, described in [46], is given in null coordinates by the Szekeres form [47]

16 $$\begin{aligned} ds^2 =-2\, \text{ e}^{-M(u)} dudv+\text{ e}^{-U(u)} \left( \text{ e}^{V(u)} dx^2 + \text{ e}^{-V(u)} dy^2 \right) \,,\nonumber \\ \end{aligned}$$

where [46]

17 $$\begin{aligned} \text{ e}^{-U}= & {} f(u)+\frac{1}{2} \,, \end{aligned}$$

18 $$\begin{aligned} \text{ e}^{V}= & {} \left( \frac{ 1+ \sqrt{ \frac{1}{2} -f}}{1-\sqrt{ \frac{1}{2}-f} } \right) ^{\lambda _1/2} \,, \end{aligned}$$

19 $$\begin{aligned} \text{ e}^{-M}= & {} kf'(u) \, \frac{ \left( \frac{1}{2}+f \right) ^{ \frac{\alpha ^2-1}{2} } }{ \left( \frac{1}{2}-f \right) ^{\alpha ^2} } \,, \end{aligned}$$

20 $$\begin{aligned} \phi= & {} \phi _0 +\frac{\lambda _2}{2} \ln \left( \frac{ 1+\sqrt{ \frac{1}{2}-f} }{ 1-\sqrt{ \frac{1}{2}-f} } \right) \,, \end{aligned}$$

and where *f*(*u*) is an arbitrary (but regular) function of *u*, $\lambda _{1,2}$ and *k* are constants, and [46]

21 $$\begin{aligned} \alpha ^2 = \frac{\lambda _1^2}{4}+\lambda _2^2 \,. \end{aligned}$$

In this case we have

22 $$\begin{aligned} \nabla _{\mu } \phi = -\frac{\lambda _2 f'(u) }{\sqrt{\frac{1}{2}-f} \left( 1+ 2 \, f \right) } \, \delta _{\mu u} \end{aligned}$$

and

23 $$\begin{aligned} \left( g^{\mu \nu } \right) =\left( \begin{array}{cccc} 0 &{} -\text{ e}^{M} &{} 0 &{} 0 \\ -\text{ e}^{M} &{} 0 &{} 0 &{}0 \\ 0 &{} 0&{} \text{ e}^{U-V} &{} 0 \\ 0 &{} 0&{} 0 &{} \text{ e}^{U+V}\\ \end{array} \right) \,, \end{aligned}$$

using which one obtains

24 $$\begin{aligned} \nabla ^c \phi \nabla _c \phi = g^{uu}\phi '(u)=0 \,. \end{aligned}$$

In this case $\ell ^c \equiv \nabla ^c \phi$ can be a null vector without contradicting the vanishing of the divergence $\nabla ^c \ell _c= \Box \phi = 0$ because we have planar, instead of spherical, symmetry and null geodesics with tangents $\ell ^c$ can form a non-expanding congruence.

## Conclusions

Vaidya geometries sourced by a massless scalar field $\phi$ acting as a null dust cannot be solutions of the Einstein equations (1). The physical reason is that these spherically symmetric geometries must necessarily have non-vanishing expansion, which contradicts the Klein–Gordon equation $\Box \phi =0$. In fact, an (irrotational) null dust created from a lighlike scalar field gradient $\ell _a=\nabla _a\phi$ must necessarily have zero divergence $\nabla ^c \ell _c=\Box \phi$, which is incompatible with the expanding (contracting) nature of the outgoing (ingoing) Vaidya geometries. By contrast, the exact plane wave (16) can be sourced by a scalar field with lightlike gradients because the requirement of zero divergence $\nabla ^c \ell _c=0$ is allowed by the planar symmetry of this geometry.

## Data Availability

This manuscript has no associated data or the data will not be deposited [Authors’ comment: This work is purely theoretical.]

## Footnotes

1 We follow the notation of Ref. [3] and use units in which the speed of light and Newton’s constant are unity.

2 This null dust can be interpreted also as an imperfect fluid with energy density, radial pressure, and heat flux density all equal, as discussed in [40].

3 Greek indices now denote tensor components in a particular coordinate system.
